# Reflectance Confocal Microscopy for Treatment Response Monitoring in Locally Advanced Basal Cell Carcinoma Treated with Sonidegib: A Prospective Observational Study

**DOI:** 10.3390/cancers18172858

**Published:** 2026-09-03

**Authors:** Federico Venturi, Carlotta Baraldi, Barbara Corti, Barbara Melotti, Elisabetta Magnaterra, Biagio Scotti, Emi Dika

**Affiliations:** 1 Department of Medical and Surgical Sciences (DIMEC), Alma Mater Studiorum University of Bologna, 40138 Bologna, Italyemi.dika3@unibo.it (E.D.); 2 Oncologic Dermatology Unit, IRCCS Azienda Ospedaliero Universitaria di Bologna, 40138 Bologna, Italy; 3 Pathology Unit, IRCCS Azienda Ospedaliero Universitaria di Bologna, 40138 Bologna, Italy; 4 Oncology Unit, IRCCS Azienda Ospedaliera-Universitaria di Bologna, 40138 Bologna, Italy

**Keywords:** basal cell carcinoma, locally advanced basal cell carcinoma, sonidegib, hedgehog inhibitors, reflectance confocal microscopy, treatment response monitoring, noninvasive imaging, neoadjuvant therapy, presurgical mapping, skin cancer

## Abstract

**Background/Objectives:** Conventional clinical and dermoscopic assessment may overestimate treatment response in locally advanced basal cell carcinoma (laBCC) treated with Hedgehog pathway inhibitors due to treatment-induced fibrosis, pigment retention, and stromal remodeling. Reflectance confocal microscopy (RCM) enables noninvasive, near-histologic resolution skin imaging and may improve response evaluation accuracy. This study evaluated the role of RCM in monitoring treatment response and guiding presurgical planning in laBCC patients treated with sonidegib. **Methods:** In this prospective, single-center, observational pilot study, 15 consecutive patients with laBCC unsuitable for upfront surgery received sonidegib 200 mg daily. Treatment response was assessed using integrated clinical examination and RCM at baseline and follow-up. Study-specific response categories (complete response [CR], partial response [PR], stable disease [SD], progressive disease [PD]) were adapted from RECIST principles. Presurgical RCM mapping was performed in partial responders. Interobserver agreement between two blinded RCM readers was assessed using Cohen’s kappa (κ). **Results:** The objective response rate was 93.3% (CR: 26.7%; PR: 66.7%; PD: 6.7%). Dose modifications were required in 73.3% of patients; adverse events occurred in 60.0%, most commonly dysgeusia (33.3%) and muscle cramps (20.0%). Clinical–RCM discordance was identified in 13.3% of patients: lesions classified as CR by conventional evaluation were reclassified as PR after RCM detected persistent basaloid tumor nests, prompting treatment continuation (Interobserver agreement: κ = 0.62–0.86). **Conclusions:** RCM identified residual disease missed by conventional assessment, directly influencing treatment decisions. These findings support integrating RCM into a multimodal, response-adapted management strategy for laBCC. However, the limited sample size warrants cautious interpretation, and larger multicenter studies are required before broad clinical implementation.

## Simple Summary

Locally advanced basal cell carcinoma (laBCC) is typically treated with Hedgehog pathway inhibitors such as sonidegib, which achieve high response rates. However, accurately assessing treatment response remains challenging, as conventional clinical and dermoscopic evaluation can both overestimate and underestimate residual disease due to treatment-induced fibrosis, inflammation, and pigment persistence. In this real-world study, sonidegib demonstrated a 93.3% objective response rate with flexible, patient-adapted dosing. Reflectance confocal microscopy (RCM) was integrated into treatment monitoring, enabling near-histologic, noninvasive visualization of residual tumor islands, confirmation of complete clearance, and differentiation of viable tumor from posttreatment changes. Discordance between conventional assessment and RCM findings directly influenced treatment decisions, supporting RCM as a valuable decision-making tool within a personalized, response-adapted management strategy for laBCC.

## 1. Introduction

Basal cell carcinoma (BCC) is the most common human malignancy, with approximately 5.4 million keratinocyte carcinomas diagnosed annually in the United States alone, of which approximately 80% are BCCs [1,2,3]. The majority of cases are amenable to surgical management. However, a subset of tumors progresses to locally advanced BCC (laBCC), characterized by extensive local invasion, involvement of critical anatomical structures, or unsuitability for standard surgical excision or radiotherapy [1,4,5,6,7,8]. Beyond classical driver mutations, emerging evidence highlights the role of microRNAs as key epigenetic regulators in the molecular pathogenesis of BCC and, more broadly, in skin cancer aggressiveness [9,10,11,12,13]. In the specific context of Hedgehog inhibitor therapy, next-generation sequencing of the BCC miRNome under neoadjuvant vismodegib identified 33 upregulated miRNAs and 39 potentially novel miRNA candidates, further expanding the landscape of non-coding RNA involvement in treatment-related tumor biology [14]. In this setting, inhibition of the Hedgehog signaling pathway has emerged as a cornerstone of treatment [15,16,17,18,19]. Among available agents, sonidegib has demonstrated significant efficacy in inducing tumor regression, improving operability, and, in selected cases, enabling a cytoreductive or neoadjuvant approach prior to surgery [20,21,22,23,24]. Approved by the FDA in 2015 for adult patients with laBCC not amenable to curative surgery or radiotherapy, sonidegib demonstrated sustained efficacy both in the pivotal BOLT trial and in real-world studies [25,26,27].

Despite these advances, a critical unmet need remains: the accurate and reproducible assessment of treatment response in laBCC during Hedgehog inhibitor therapy [28]. Treatment-induced fibrosis, pigment retention, vascular remodeling, and architectural distortion may reduce the reliability of conventional clinical and dermoscopic assessment after Hedgehog inhibitor therapy. Consequently, residual disease may be either overestimated or underestimated, depending on the predominant post-treatment changes, highlighting the need for complementary imaging modalities capable of improving response assessment [29,30].

Notably, half of clinically complete-responding tumors treated with Hedgehog inhibitors have been shown to still harbor residual tumor on imaging and histopathology [29,31]. While radiologic criteria such as RECIST (Response Evaluation Criteria in Solid Tumors) criteria are widely adopted in oncology to classify responses (complete response [CR], partial response [PR], stable disease [SD], or progressive disease [PD]), their applicability to cutaneous tumors remains limited [22,32,33]. RECIST relies on dimensional changes that may not reflect the true biological response of tumors with infiltrative growth patterns or subclinical extension [33]. Notably, the pivotal BOLT trial employed modified RECIST criteria incorporating MRI, clinical photography, and histopathology, acknowledging that standard RECIST is inadequate for laBCC due to ulceration, cyst formation, scarring, and ill-defined borders [20,34]. In clinical practice, especially when sonidegib is used in a neoadjuvant or cytoreductive setting, the determination of residual tumor burden and precise surgical margins is crucial [21,25,31,35]. However, there is currently no standardized, noninvasive method to guide the timing of surgery or to confirm complete tumor clearance [36]. As a result, assessment often relies on clinical and dermoscopic evaluation, sometimes supplemented with biopsy, even in cases with apparent clinical regression [29,30]. This approach, while reliable, is invasive, subject to sampling error, with limited biopsy sampling failing to identify deeper or aggressive tumor components in approximately 1 in 6 cases, and may not adequately capture the heterogeneity of residual disease [37,38].

Noninvasive imaging techniques have the potential to bridge this gap [36,39,40]. Among these, reflectance confocal microscopy (RCM) allows in vivo, real-time visualization of the epidermis and superficial dermis at near-histologic resolution. In BCC, RCM enables the identification of key diagnostic features including tumor islands with peripheral palisading, cleft-like dark spaces, increased dermal vasculature, and stromal changes such as bright collagen bundles and inflammatory infiltrates [41,42,43,44]. RCM has already shown utility in diagnosis, margin delineation, and monitoring of nonmelanoma skin cancers, including treatment response assessment following topical therapies and photodynamic therapy [29,39,40,45,46,47]. However, its role in dynamically assessing treatment response to Hedgehog inhibitors and in guiding surgical decision-making remains insufficiently defined, and challenges persist in distinguishing treatment-induced changes such as fibrosis, nuclear streaming, and vascular alterations from true residual disease [29,30,36,48].

In this context, the present study aimed to evaluate the role of RCM as a non-invasive adjunct to clinical assessment for monitoring treatment response and supporting presurgical planning in patients with laBCC treated with sonidegib. Specifically, we explored its potential to identify residual superficial disease and support therapeutic decision-making in a setting where standardized non-invasive response assessment is currently lacking.

To the best of our knowledge, this is the first prospective study specifically evaluating the role of RCM for treatment response assessment in patients with locally advanced basal cell carcinoma treated with Sonidegib. In particular, we investigated whether RCM could improve the detection of residual disease beyond conventional clinical and dermoscopic assessment and support therapeutic decision-making during routine clinical practice.

The present study was therefore designed to provide preliminary clinical data that may support and inform future larger multicenter investigations.

## 2. Materials and Methods

We conducted a prospective observational pilot study including consecutive patients diagnosed with laBCC who were treated with sonidegib at the Oncologic Dermatology Unit of IRCCS Azienda Ospedaliero-Universitaria of Bologna, Policlinico of Sant’Orsola between January 2024 and December 2025. Patients were considered eligible if their tumors were deemed unsuitable for upfront surgery and/or radiotherapy due to size, location, or anticipated functional or cosmetic impairment via multidisciplinary assessment. Because laBCC requiring systemic therapy is an uncommon clinical condition managed in specialized referral centers, no formal sample size calculation was performed. Instead, all consecutive eligible patients treated during the study period were prospectively included, reflecting real-world clinical practice. Clinical, demographic, and treatment-related data were collected from a prospectively maintained institutional database. All patients provided informed consent for treatment and data collection, in accordance with institutional and ethical standards. All patients were treated with sonidegib at a standard dose of 200 mg once daily.

Patients underwent clinical, dermoscopic, and RCM evaluations at baseline and at monthly intervals throughout treatment. Additional visits were scheduled whenever clinically indicated because of treatment-related adverse events or suspected disease progression. Although response evolution was assessed at each visit, the best overall response reported in this study was assigned at the last treatment assessment immediately before treatment discontinuation or, in patients undergoing planned cytoreductive/neoadjuvant surgery, immediately before surgical resection.

RCM examinations were performed using a commercially available confocal microscope (VivaScope® 1500/3000, Caliber Imaging & Diagnostics, Rochester, NY, USA), which provides noninvasive, real-time imaging of the skin at near-histologic resolution. Imaging was performed using multiple adjacent mosaics covering the clinically visible lesion and surrounding skin. Images were acquired from the stratum corneum to the superficial dermis (approximately 200–250 μm depth), enabling evaluation of the epidermis, dermoepidermal junction, and superficial dermis for residual tumor structures and treatment-induced changes.

Following treatment completion, patients underwent clinical, dermoscopic, and RCM evaluations every 3 months during follow-up, with additional assessments performed whenever clinically indicated according to individual patient needs or in cases of suspected recurrent disease. Dose modifications, including alternate-day administration, intermittent dosing, or temporary/permanent discontinuation, were implemented at the discretion of the treating physician based on the occurrence and severity of treatment-related adverse events.

Treatment response was assessed through integrated clinical evaluation and RCM at baseline and during follow-up. Given the absence of validated RCM-based response criteria for Hedgehog inhibitor therapy in laBCC, study-specific response categories were defined as follows, adapted from RECIST principles to the cutaneous setting:

• Complete response (CR): absence of clinically and RCM-detectable tumor;
• Partial response (PR): reduction in tumor extent with persistence of residual RCM features;
• Stable disease (SD): no significant change in tumor features;
• Progressive disease (PD): increase in tumor extent or emergence of new tumor areas.

Particular attention was given to the persistence or disappearance of tumor islands and associated architectural features, which were considered the most reliable indicators of residual disease.

RCM evaluation focused on established BCC criteria: tumor islands/nests; peripheral palisading; peritumoral clefting; dark silhouettes; increased vascularization; inflammatory infiltrate; stromal fibrosis [48]. RCM response assessment was based on the same predefined diagnostic criteria used at baseline. During follow-up, image interpretation was performed through direct comparison with each patient’s baseline examination, allowing differentiation between persistent tumor structures and treatment-induced changes such as fibrosis, collagen remodeling, and inflammatory infiltrates.

Patients achieving a PR were considered for surgical management within a cytoreductive/neoadjuvant approach [21,31,35,49]. In these cases, presurgical tumor mapping was performed using RCM to identify residual tumor foci and delineate subclinical margins [41,42].

### 2.1. Interobserver Agreement

To reduce assessment bias, RCM image evaluation was performed independently by two experienced confocal microscopy readers (F.V. and E.D.). Readers were blinded to clinical outcomes, treatment duration, and prior response assessments. Discrepancies were resolved through consensus review; in cases of persistent disagreement, a third senior evaluator adjudicated the final interpretation.

### 2.2. Statistical Analysis

Descriptive statistics summarized patient characteristics and outcomes. The objective response rate (ORR) was defined as CR + PR, and the disease control rate (DCR) as CR + PR + SD. Interobserver agreement was assessed using unweighted Cohen’s kappa. Stromal fibrosis and inflammatory infiltrates were evaluated as a combined category of treatment-induced stromal alterations because these findings frequently coexist following Hedgehog inhibitor therapy and are interpreted together in the overall assessment of treatment response. Exploratory analyses evaluated associations between adverse events, dose modifications, and response. Due to the small sample size, analyses were considered descriptive and hypothesis-generating.

## 3. Results

A total of 15 patients with laBCC were included. Baseline demographic, clinical, and tumor characteristics are summarized in Table 1.

All tumors were considered unsuitable for upfront surgical management due to size, anatomical location, or anticipated functional and cosmetic morbidity via multidisciplinary evaluation. All patients started treatment with sonidegib 200 mg once daily. During follow-up, dose modifications were required in 73% of patients, mainly due to treatment-related AEs. The most frequent adjustments included alternate-day dosing, with some patients requiring further reduction to intermittent regimens. One patient (6.7%) permanently discontinued treatment due to toxicity and disease progression. The median follow-up duration for the study cohort was 14.7 months (IQR 12.1–17.9). Most AEs were mild to moderate (Grade 1–2) and manageable with dose modification. Table 1.

**Figure 1:**
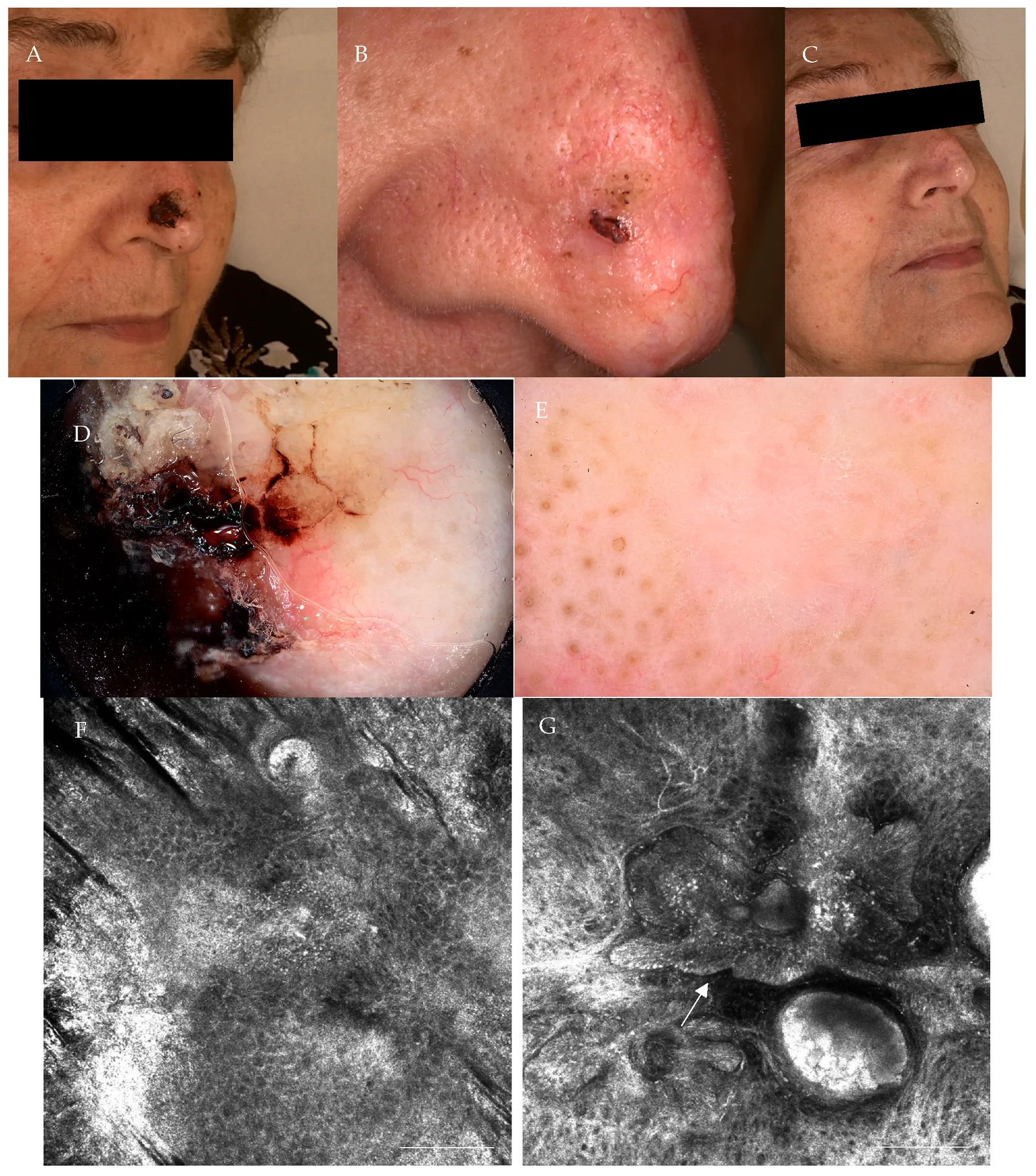
Clinical and reflectance confocal microscopy (RCM) evolution during sonidegib treatment in locally advanced basal cell carcinoma of the nose. (**A**) Clinical appearance at baseline (T0); (**B**) after 3 months of sonidegib treatment (T3); (**C**) after 6 months of treatment (T6), showing apparent clinical complete response. (**D**) Dermoscopic examination at baseline demonstrated arborizing vessels of varying caliber, focal ulceration covered by hemorrhagic crusts, white structureless/fibrotic areas on a predominantly pink-white background; (**E**) Post-treatment dermoscopy showing disappearance of the characteristic dermoscopic features of basal cell carcinoma and persistence of subtle white scar-like areas consistent with post-treatment remodeling. Reflectance confocal microscopy performed during the same visit identified residual microscopic disease, leading to reclassification from complete response to partial response. (**F**) RCM image (0.5 × 0.5 mm) acquired within the treated area at the epidermal layer and (**G**) at the dermoepidermal junction showing a lobulated bright tumor island that is well demarcated by surrounding cleft-like dark spaces (→) despite complete clinical regression, indicating residual disease and supporting reclassification from complete response to partial response. Scale bar = 100 μm.

**Figure 2:**
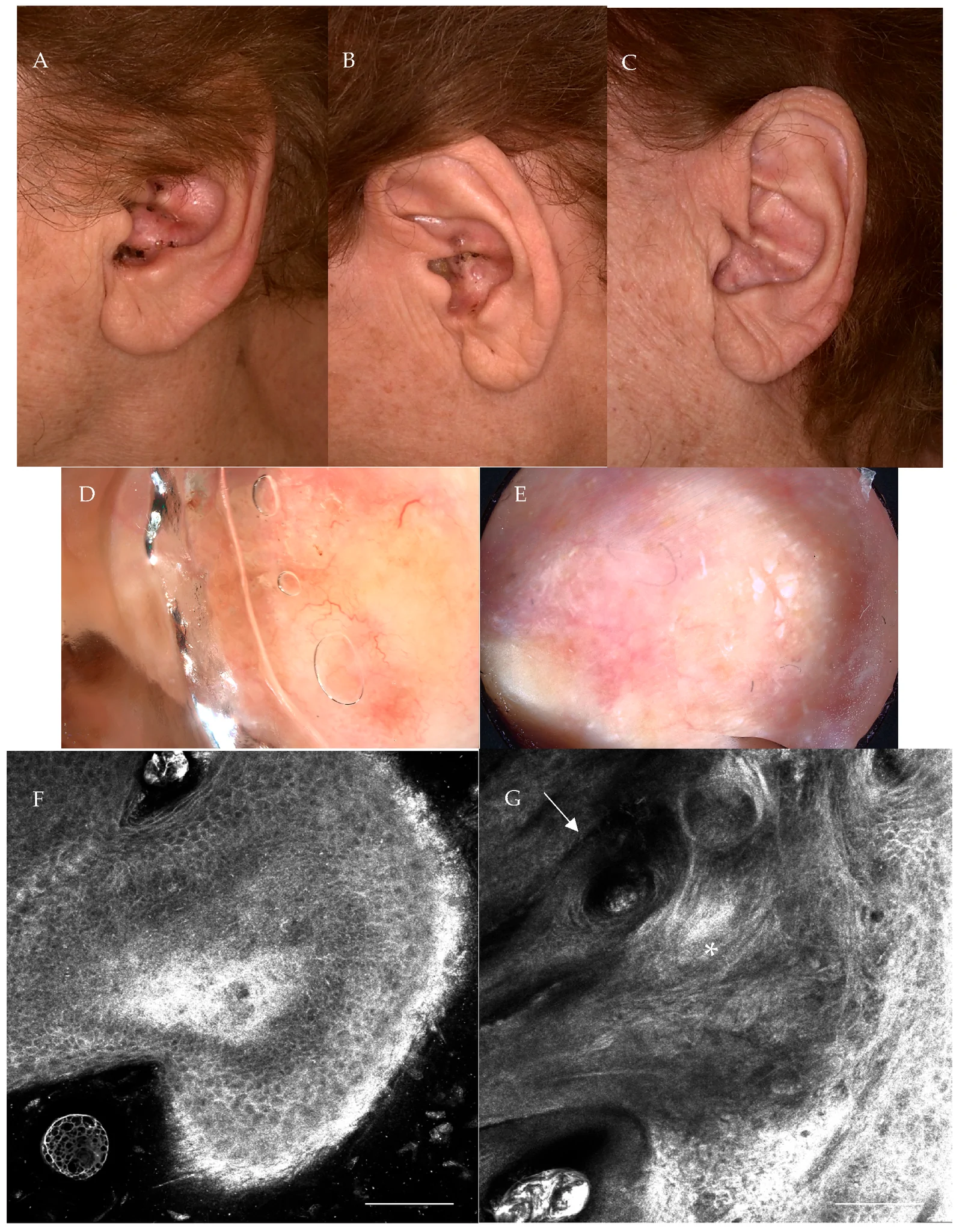
Clinical and reflectance confocal microscopy (RCM) evolution during sonidegib treatment in locally advanced basal cell carcinoma of the ear. (**A**) Clinical appearance at baseline (T0); (**B**) after 3 months of sonidegib treatment (T3); (**C**) after 6 months of treatment (T6), showing apparent clinical complete response. (**D**) Baseline dermoscopy of the auricular concha showing pink-white structureless areas with fine arborizing telangiectatic vessels and focal superficial erosions, consistent with basal cell carcinoma. (**E**) Post-treatment dermoscopic image after sonidegib therapy showing apparent complete dermoscopic response. The characteristic dermoscopic features of basal cell carcinoma are no longer detectable, with persistence of subtle white scar-like areas and faint telangiectatic vessels compatible with post-treatment fibrosis and tissue remodeling. Reflectance confocal microscopy performed during the same visit subsequently identified residual microscopic tumor nests, resulting in reclassification from complete response to partial response. (**F**) RCM image at the epidermal level showing absence of residual tumor structures. (**G**) RCM image at the dermoepidermal junction depicts tumor islands surrounded by thickened collagen bundles (*). There are canalicular vessels with bright cells characteristic of dilated, telangiectatic vessels in the surrounding dermis (→), supporting reclassification from complete response to partial response. Scale bar = 100 μm.

Response was assessed through integrated clinical and RCM evaluation. The ORR was 93.3% (14/15 patients), including CR: 4 patients (26.7%); PR: 10 patients (66.7%); SD: 0 patients (0%); PD: 1 patient (6.7%). A discordance between clinical/dermoscopic and RCM assessment was observed in 2 out of 15 patients (13.3%). In both cases, lesions were classified as CR based on clinical and dermoscopic evaluation, while RCM examination performed during the same visit revealed persistent basaloid tumor nests at the dermoepidermal junction, indicating residual disease (Figure 1 and Figure 2). These findings led to reclassification of response from CR to PR and prompted continuation of sonidegib therapy beyond the time point of apparent clinical clearance. Results are fully displayed in Table 2.

Interobserver agreement for RCM evaluation between two independent, blinded readers was substantial to excellent. Tumor islands/nests: κ = 0.84; Peripheral palisading: κ = 0.78; Peritumoral clefting: κ = 0.75; Stromal fibrosis: κ = 0.62 and inflammatory infiltrate: κ = 0.70. Overall response classification (CR, PR, PD) showed excellent reproducibility (κ = 0.86). Discrepancies were infrequent and resolved by consensus. The comparatively lower agreement observed for stromal and inflammatory features likely reflects the greater subjectivity of interpreting treatment-induced fibrosis, collagen remodeling, and inflammatory infiltrates, which may partially overlap with post-treatment reparative changes. In contrast, classical tumor-related criteria such as basaloid tumor islands and peripheral palisading demonstrated excellent reproducibility.

## 4. Discussion

In this real-world cohort of patients with laBCC, treatment with sonidegib resulted in a high ORR (93.3%), with more than 25% of patients achieving CR. Notably, this level of efficacy was maintained despite frequent dose modifications, supporting the feasibility of flexible, patient-adapted dosing strategies in routine clinical practice [25,27]. The efficacy observed in our cohort is consistent with outcomes reported in pivotal clinical trials and real-world series [25,27,50].

A major challenge in the management of laBCC treated with Hedgehog inhibitors lies in the accurate evaluation of treatment response [28]. Increasing evidence indicates that conventional methods may be unreliable in this setting [29,51].

These observations are consistent with the findings of Couzan et al., who compared clinical examination, dermoscopy, and RCM during and after vismodegib therapy and demonstrated the markedly higher sensitivity of RCM for detecting residual BCC. Specifically, RCM achieved a sensitivity of 95%, compared with 35% for dermoscopy and 33% for clinical examination, highlighting its ability to identify subclinical residual disease not detectable by conventional assessment [30]. In this scenario, the present prospective study focused on longitudinal response monitoring in patients with la BCC treated with sonidegib in a real-world setting.

A recent real-world study demonstrated that post-treatment changes induced by sonidegib, including fibrosis, vascular remodeling, and persistent pigmentation, can lead to a high rate of false-positive assessments, even in lesions with complete histopathological clearance [51]. In particular, dermoscopic features traditionally associated with BCC, such as arborizing vessels or blue-gray structures, may persist after tumor eradication due to pigment-laden macrophages and stromal alterations, rather than viable tumor tissue [52,53,54]. This phenomenon has been described as a tumor–pigment regression desynchrony, in which tumor cell clearance precedes the resolution of pigment and inflammatory changes. As a result, lesions may remain clinically and dermoscopically suspicious despite the absence of residual tumor, creating a critical diagnostic gap and potentially leading to overtreatment or unnecessary invasive procedures [29,51,55,56].

Conversely, substantial superficial tumor regression may also conceal persistent microscopic disease, resulting in false-negative evaluations. Our findings exemplify this scenario. In two patients, lesions considered to have achieved CR on clinical and dermoscopic examination were reclassified as PR after RCM demonstrated persistent basaloid tumor nests during the same visit. In these cases, lesions were initially classified as CR based on conventional noninvasive evaluation, while RCM detected persistent tumor nests at the dermoepidermal junction, indicating residual disease. Indeed, RCM findings directly influenced clinical management by prompting extension of systemic therapy, thereby potentially preventing premature treatment discontinuation and incomplete tumor clearance.

This observation further supports the limitations of clinical and dermoscopic assessment after Hedgehog inhibitor therapy, as previously reported, and underscores the risk of overestimating CR when relying solely on these modalities [30]. Importantly, interobserver agreement for RCM evaluation was consistent with prior studies reporting observed agreement of 82–84% for BCC presence and excellent intraobserver reliability (κ = 0.909) [57].

Within this context, the integration of RCM represents a key strength and innovative aspect of our study. Importantly, the principal finding of the present study is not that RCM establishes definitive tumor clearance, but that it identified residual microscopic disease in lesions that appeared to have achieved CR on clinical and dermoscopic examination, directly influencing therapeutic decision-making. Whether RCM-confirmed CR accurately predicts complete histopathologic clearance or long-term local control remains to be established in larger prospective studies with systematic histopathologic correlation and longer follow-up.

Taken together, our results suggest that the management of laBCC with Hedgehog inhibitors should evolve toward a personalized, response-adapted approach, integrating flexible dosing strategies to improve tolerability, advanced noninvasive imaging for accurate response assessment and selective surgical intervention in partial responders. Within this framework, RCM may serve as a noninvasive bridge between clinical evaluation and histopathology, reducing diagnostic uncertainty and potentially limiting the need for repeated biopsies.

This study has several limitations. First, the small sample size (*n* = 15) limits the generalizability of the findings. However, this should be interpreted in the context of the rarity of patients with laBCC requiring systemic Hedgehog inhibitor therapy. Importantly, to the best of our knowledge, no previous prospective study has specifically investigated the role of RCM for treatment response assessment during sonidegib therapy. We therefore believe that these data provide an important initial contribution to the field and may serve as a foundation for future multicenter prospective studies. Second, although the median follow-up was 14.7 months, longer surveillance is required to determine whether RCM-based response assessment predicts durable local control and recurrence-free outcomes.

Histopathologic confirmation was available only for patients who subsequently underwent surgery after PR within the planned cytoreductive/neoadjuvant strategy and therefore could not be systematically compared with RCM findings across the entire cohort. Likewise, patients achieving RCM-confirmed CR did not routinely undergo surgical excision, precluding universal histopathologic validation. Consequently, the present study was not designed to determine the diagnostic accuracy of RCM against histopathology, but rather to evaluate its potential clinical utility as a non-invasive adjunct for treatment monitoring and therapeutic decision-making.

Moreover, the imaging depth of RCM is restricted to approximately 200–250 μm, encompassing the epidermis, DEJ, and superficial dermis. This means that deeper tumor components, particularly relevant in infiltrative, morpheaform, or micronodular BCC subtypes, cannot be reliably assessed by RCM alone. In the context of laBCC, where deep invasion is common, RCM findings should be interpreted as complementary to, rather than a replacement for, histopathologic and radiologic assessment. In the presented study, all included lesions demonstrated unequivocal diagnostic RCM features at baseline, allowing subsequent examinations to be directly compared with each patient’s pretreatment confocal images. Treatment response was therefore determined by the evolution of baseline confocal findings, including the persistence, reduction, or disappearance of basaloid tumor islands and associated morphological criteria, rather than by assessment of the entire vertical tumor extent. Accordingly, the role of RCM in this setting is to monitor superficial residual disease and support surgical decision-making by identifying residual superficial disease after Hedgehog inhibitor therapy, while assessment of deep tumor extension remains beyond its intrinsic imaging capability and should be complemented by histopathologic and, when appropriate, radiologic evaluation. In addition, treatment-induced fibrosis and inflammation may alter the optical properties of tissue, potentially reducing the diagnostic accuracy of RCM in the posttreatment setting. Third, RCM requires specialized equipment and trained operators, which may limit its widespread adoption outside tertiary referral centers.

## 5. Conclusions

As the first prospective study investigating RCM for treatment response assessment in patients with laBCC treated with sonidegib, this study provides preliminary evidence supporting the potential clinical value of RCM in treatment monitoring. Given the rarity of this patient population and the limited sample size, these findings require confirmation in larger multicenter prospective studies before they can be generalized to routine clinical practice.

## Figures and Tables

**Table 1:** Baseline characteristics and treatment exposure of patients with locally advanced basal cell carcinoma treated with sonidegib. Data are presented as number (percentage) unless otherwise indicated. All patients initiated treatment with sonidegib 200 mg once daily. Dose modifications included alternate-day dosing and further intermittent regimens according to tolerability.

Variable	Overall (*n* = 15)
Age, years, median (IQR)	79.0 (71.0–85.5)
Male Sex, *n* (%)	9 (60%)
Gorlin syndrome, *n* (%)	1 (6.7%)
Baseline tumor diameter, cm, median (IQR)	4.0 (2.1–5.9)
Tumor location, *n* (%)	
Nose	1 (6.7)
Ear	1 (6.7)
Periocular region	2 (13.3)
Other facial sites	3 (20.0)
Trunk	7 (46.7)
Upper extremity	1 (6.7)
Histopathological subtype, *n* (%)	
Nodular	5 (33.3)
Superficial	5 (33.3)
Morpheaform	3 (20.0)
Infiltrative	1 (6.7)
Micronodular	1 (6.7)
Dose modification, *n* (%)	11 (73.3%)
Alternate-day dosing	9 (60.0%)
Further reduction (intermittent regimens)	2 (13.3%)
Permanent discontinuation	1 (6.7%)
Follow-up duration, months, median (IQR)	14.7 (12.1–17.9)

**Table 2:** Treatment outcomes, safety profile, and reflectance confocal microscopy (RCM) findings in patients with locally advanced basal cell carcinoma treated with sonidegib. Response was assessed through integrated clinical and RCM evaluation. The objective response rate (ORR) was defined as complete response (CR) plus partial response (PR), while the disease control rate (DCR) included CR, PR, and stable disease (SD). Adverse events (AEs) are reported as the number and percentage of patients experiencing each event. RCM findings describe the evolution of tumor and stromal features during treatment.

Outcome	Overall (*n* = 15)
**Best overall response, ** ***n*** ** (%)**	
Complete response (CR)	4 (26.7%)
Partial response (PR)	10 (66.7%)
Stable disease (SD)	0 (0%)
Progressive disease (PD)	1 (6.7%)
**Objective response rate (ORR)**	14 (93.3%)
**Disease control rate (DCR)**	14 (93.3%)
**Adverse events (any), ** ***n*** ** (%)**	9 (60.0%)
**Type of adverse events, ** ***n*** ** (%)**	
Dysgeusia	5 (33.3%)
Muscle cramps	3 (20.0%)
CPK increase	2 (13.3%)
Weight loss	2 (13.3%)
Asthenia	1 (6.7%)
Diarrhea	1 (6.7%)
**RCM findings during treatment**	Interobserver agreement (κ)
Tumor islands	0.84
Palisading	0.78
Clefting	0.75
Stromal fibrosis	0.62
Inflammatory infiltrate	0.70
Overall response classification	0.86

## Data Availability

The original contributions presented in this study are included in the article. Further inquiries can be directed to the corresponding author.

## Abbreviations

The following abbreviations are used in this manuscript:

BCC	Basal Cell Carcinoma
laBCC	Locally Advanced Basal Cell Carcinoma
CR	Complete Response
PR	Partial Response
SD	Stable Disease
PD	Progressive Disease
RCM	Reflectance Confocal Microscopy
AEs	Adverse Events
ORR	Objective Response Rate
DCR	Disease Control rate
